# Supplementary material for: Comparative multiomics analysis of cell physiological state after culture in a basket bioreactor
Source: Sci Rep. 2022 Nov 23;12:20161. doi: 10.1038/s41598-022-24687-4 (PMC9686226; doi:10.1038/s41598-022-24687-4)

6045.1:100217896:100218036:+@NW_023666045.1:100217896:100218538:+@NW_023666045.1:100219680:10[illegible]

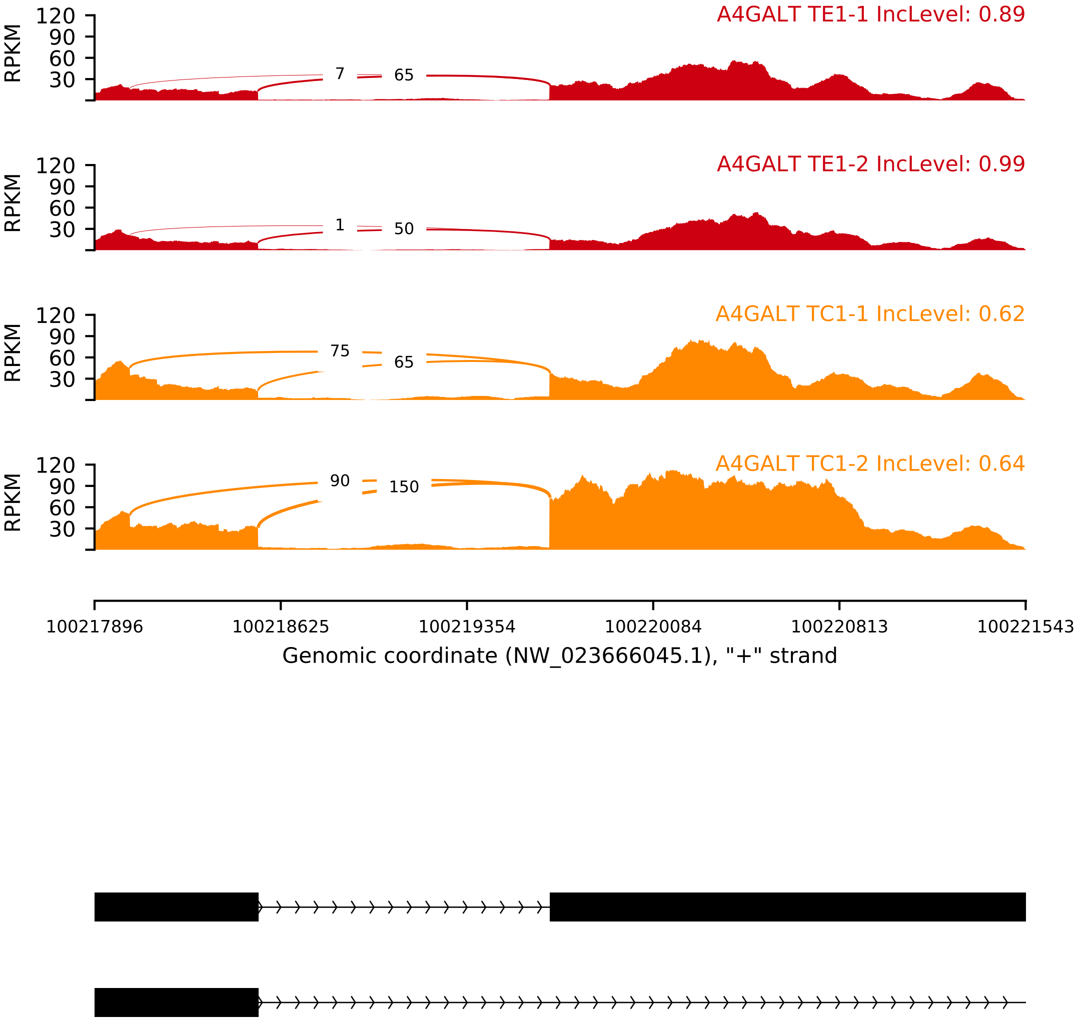

Supplement: Supplementary file 1 — Supplementary Information 1. [file 41598_2022_24687_MOESM1_ESM.zip › raw data/Transcriptomic raw data/7.AS/2.dasplot/TE1vsTC1/A5SS/6_A4GALT_NW_023666045.1_100217896_100218036_+@NW_023666045.1_100217896_100218538_+@NW_023666045.1_100219680_100221544_+.pdf]
